# Visual field deficits after epilepsy surgery: a new quantitative scoring method

**DOI:** 10.1007/s00701-018-3525-9

**Published:** 2018-04-05

**Authors:** Rick H. G. J. van Lanen, M. C. Hoeberigs, N. J. C. Bauer, R. H. L. Haeren, G. Hoogland, A. Colon, C. Piersma, J. T. A. Dings, O. E. M. G. Schijns

**Affiliations:** 1 Faculty of Health Medicine and Life Sciences, Maastricht University, Maastricht, The Netherlands; 2 Department of Neurosurgery, Maastricht University Medical Centre, PO box 5800, 6202 AZ Maastricht, The Netherlands; 3 Department of Radiology, Maastricht University Medical Centre, Maastricht, The Netherlands; 4 Department of Ophthalmology, Maastricht University Medical Centre, Maastricht, The Netherlands; 5 Academic Centre for Epileptology, Maastricht University Medical Centre and Kempenhaeghe, Maastricht/Heeze, The Netherlands; 6 School for Mental Health and Neuroscience (MHeNs), Maastricht University, Maastricht, The Netherlands

**Keywords:** Visual field deficits, Perimetry, Quadrantanopia, Epilepsy, Temporal lobectomy

## Abstract

**Background:**

Anterior temporal lobectomy (ATL) as a treatment for drug-resistant temporal lobe epilepsy (TLE) frequently causes visual field deficits (VFDs). Reported VFD encompasses homonymous contralateral upper quadrantanopia. Its reported incidence ranges from 15 to 90%. To date, a quantitative method to evaluate postoperative VFD in static perimetry is not available. A method to quantify postoperative VFD, which allows for comparison between groups of patients, was developed.

**Methods:**

Fifty-five patients with drug-resistant TLE, who underwent ATL with pre- and postoperative perimetry, were included. Temporal lobe resection length was measured on postoperative MRI. Percentage VFD was calculated for the total visual field, contralateral upper quadrant, or other three quadrants combined.

**Results:**

Patients were divided into groups by resection size (< 45 and ≥ 45 mm) and side of surgery (right and left). We found significant higher VFD in the ≥ 45 vs. < 45 mm group (2.3 ± 4.4 vs. 0.7 ± 2.4%,*p* = 0.04) for right-sided ATL. Comparing VFD in both eyes, we found more VFD in the right vs. left eye following left-sided ATL (14.5 ± 9.8 vs. 12.9 ± 8.3%, *p* = 0.03). We also demonstrated significantly more VFD in the < 45 mm group for left- vs. right-sided surgery (6.7 ± 6.7 vs. 13.1 ± 7.0%, *p* = 0.016). A significant quantitative correlation between VFD and resection size for right-sided ATL was shown (*r* = 0.52, *p* < 0.01).

**Conclusions:**

We developed a new quantitative scoring method for the assessment of postoperative visual field deficits after temporal lobe epilepsy surgery and assessed its feasibility for clinical use. A significant correlation between VFD and resection size for right-sided ATL was confirmed.

## Introduction

Despite the availability of many antiepileptic drugs, an estimated 30–40% of epilepsy patients are drug-resistant [5, 7]. In selected patients, epilepsy surgery is a successful and cost-effective therapy to achieve seizure freedom [6]. Epilepsy surgery for temporal lobe epilepsy has been found particularly effective with reported seizure freedom rates of 70–80% [20]. Temporal lobe surgery encompasses anterior temporal lobectomy (ATL) with or without amygdalohippocampectomy or tailored variants. Complications related to ATL include, among others, visual field deficits (VFDs), cognitive complaints, neurological deficits and infections [2]. The most common reported VFD is a contralateral homonymous upper quadrantanopia, clinically often referred to as a pie in the sky [6, 23].

The reported incidence of postoperative VFD widely ranges from 15 up to 90% [1, 13, 14, 21, 26]. This deficit is caused by damage to the anterior part of the optic radiation extending from the lateral geniculate body of the thalamus into the anterior part of the temporal lobe, on its way to the visual cortex. This anterior bending of the optic radiation in the temporal lobe is also known as “Meyer’s loop” (ML) [19, 31]. The distance between the most anterior part of Meyer’s loop and the temporal pole varies widely between individuals [1, 24, 31]. Recent reports have estimated a temporal pole to Meyer’s loop distance variation of 22–44 mm [1, 24, 31]. As temporal lobe resections for epilepsy surgery may extend up to 90 mm, Meyer’s loop can easily be injured [15]. Nevertheless, there is controversy on whether the size of resection correlates with the size of visual field deficit [11, 21].

In current literature, there is no consensus about the method to determine the extent of VFD following ATL. Moreover, various perimetry methods to assess VFD are available. A few studies have reported on the relation between the extent of VFD and size of resection based on magnetic resonance imaging (MRI), differentiating perimetric data into broad categories [15, 16, 21, 29]. Barton et al. [1] suggested a method to estimate postsurgical VFD quantitatively using Goldmann perimetry, a kinetic perimetry method. However, there is no quantitative method available to evaluate VFD in static perimetry.

Therefore, we aimed to develop a method to quantify the postoperative VFD after temporal lobe surgery and propose a new quantitative scoring method independent of the perimetry procedure. Using this method, we assessed the relation between length of temporal lobe resection and postsurgical VFD and compared differences between right- and left-sided surgeries.

## Materials and methods

This study was conducted at the Maastricht University Medical Centre (MUMC+), Maastricht, The Netherlands. This university hospital is a tertiary referral and expertise centre for epileptology and epilepsy surgery. This study complies with the Declaration of Helsinki and principals of Good Clinical Practice. Informed consent was obtained from all individual participants before they were included in this study.

### Patients

Patients with drug-resistant temporal lobe epilepsy (TLE) who underwent ATL between 2000 and 2014 with pre- and postoperative MRI and perimetry were selected from a prospectively collected database. From this database, patient inclusion and data analyses were performed retrospectively. Included patients were mentally competent adults, who were candidates for epilepsy surgery because of chronic drug-resistant TLE, as was revealed by thorough preoperative examination. Surgical treatment was an anterior temporal lobectomy with or without amygdalohippocampectomy. None of the included patients underwent temporal lobe surgery before.

Medical charts, pre- and postoperative MRI scans of the brain, surgical reports and pre- and postoperative perimetry of eligible patients were reviewed. All patients underwent a pre- and postoperative MRI scan of the brain to determine the exact resection length, and perimetry to compare visual field deficits. Postoperative MRI scan and perimetry were performed at the 3-month outpatient follow-up visit to limit the confounding effect of postoperative oedema, blood, or cerebrospinal fluid collection.

### Imaging

**Fig. 1:**
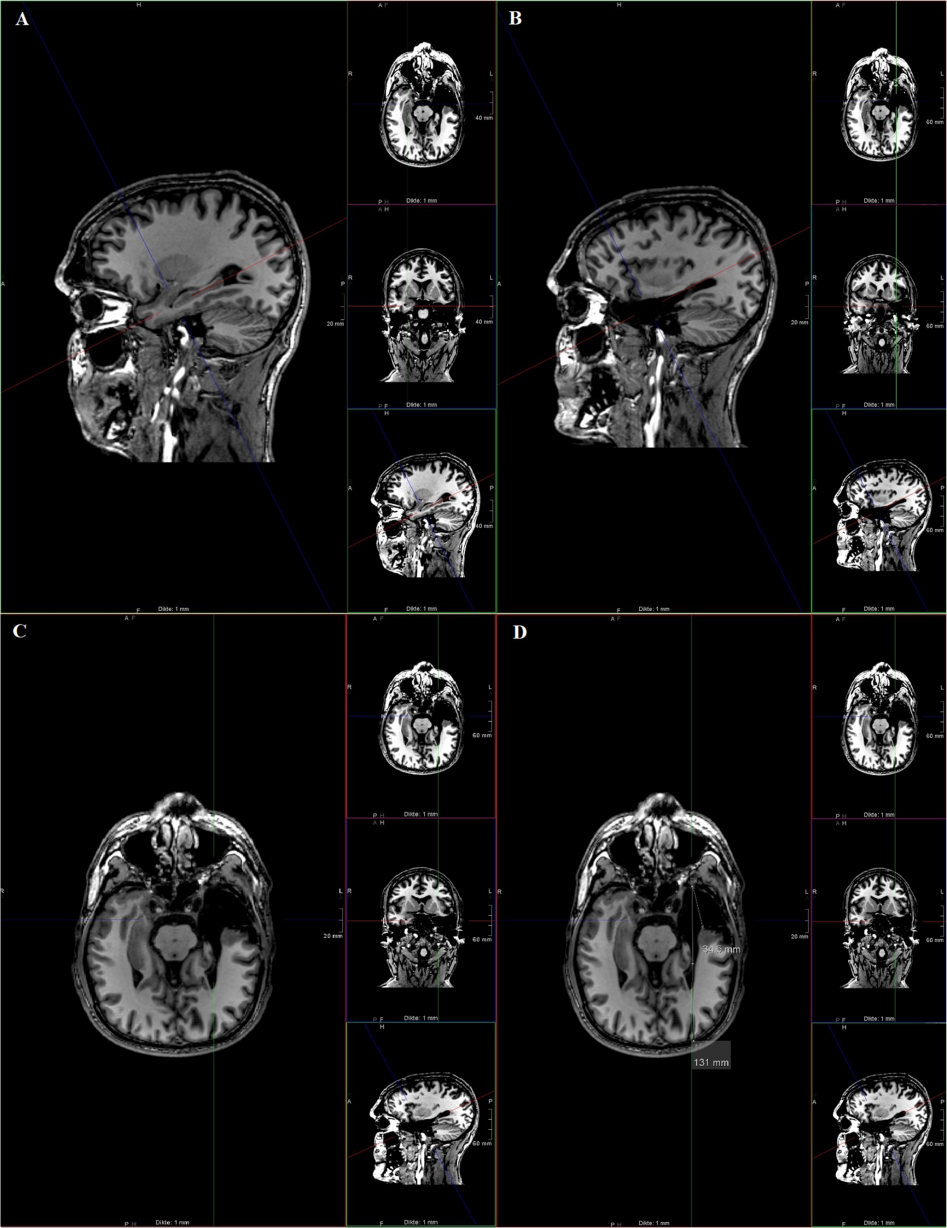
To measure the extent of resection, we reconstructed the 3D T1 scan in a plane parallel to the hippocampus at the non-resected side, as shown in **a**. Next, we returned to the resected side within the same plane (**b**) and switched back to the axial view (**c**). Finally, the anterior-posterior (AP) length of resection was estimated by measuring the distance from the anterior tip of the middle sphenoid fossa (which had contained the resected temporal pole) to the posterior margin of the resection cavity in axial view. To compensate for variations in head size and possible metrical distortion introduced by MRI, the extent of resection was expressed as a fraction of the distance between the anterior tip of the middle sphenoid fossa to the occipital pole (anterior temporal-occipital pole, or ATOP distance), shown in **d**

MRI scans were obtained in standard fashion with axial T2, fluid attenuation inversion recovery (FLAIR), fast field echo (FFE), coronal FLAIR and inversion recovery (IR) images and a 3D T1. The 3D T1-weighted dataset was used for determining the length of resection. The 3D T1 typically consisted of about 160 slices, with time to repeat (TR) of 8 ms, time to echo (TE) of 4 ms, flip angle of 8°, matrix of 240 × 240 mm^2^, number of excitations (NEX) of 1 and voxel size of 1 × 1 × 1 mm^3^. The method to measure the extent of resection is shown in Fig. 1.

Postoperative MRI was carefully reviewed by a trained neuro-radiologist (MH) with experience in the radiological assessment of epilepsy patients. The radiologist was blinded to the intended resection size and postoperative VFD. The anterior-posterior (AP) length of resection and the distance between the anterior tip of the middle sphenoid fossa to the occipital pole (anterior temporal-occipital pole, or ATOP distance) were measured. Since the distance from the anterior tip of ML to the temporal pole ranges between 22 and 44 mm, we divided patients into two groups by resection length: less than 45 mm or equal to or greater than 45 mm, as shown in Table 1.

**Table 1:** Patient and surgery characteristics for resection length groups

Characteristic	Resection size on postoperative MRI		*p* value
	< 45 mm No. (%), *n* = 30	≥ 45 mm No. (%), *n* = 25	
Male	12 (40.0)	13 (52.0)	0.37
Age (mean, range)	38 (17–59)	40 (15–62)	0.62
Surgery			
Right-sided	14 (56.0)	11 (44.0)	0.84
Resection size (AP) (mean, range)	36.1 (11–43)	50.4 (45–80)	< 0.01
ATOP (mean, range)	124.9 (117–134)	127.9 (116–138)	0.08
Ratio AP-ATOP (mean, range)	0.29 (0.09–0.37)	0.39 (0.34–0.65)	< 0.01

*AP* anterior-posterior resection distance in millimetres, *ATOP* anterior temporal-occipital pole distance in millimetres

### Perimetry scoring method

**Fig. 2:**
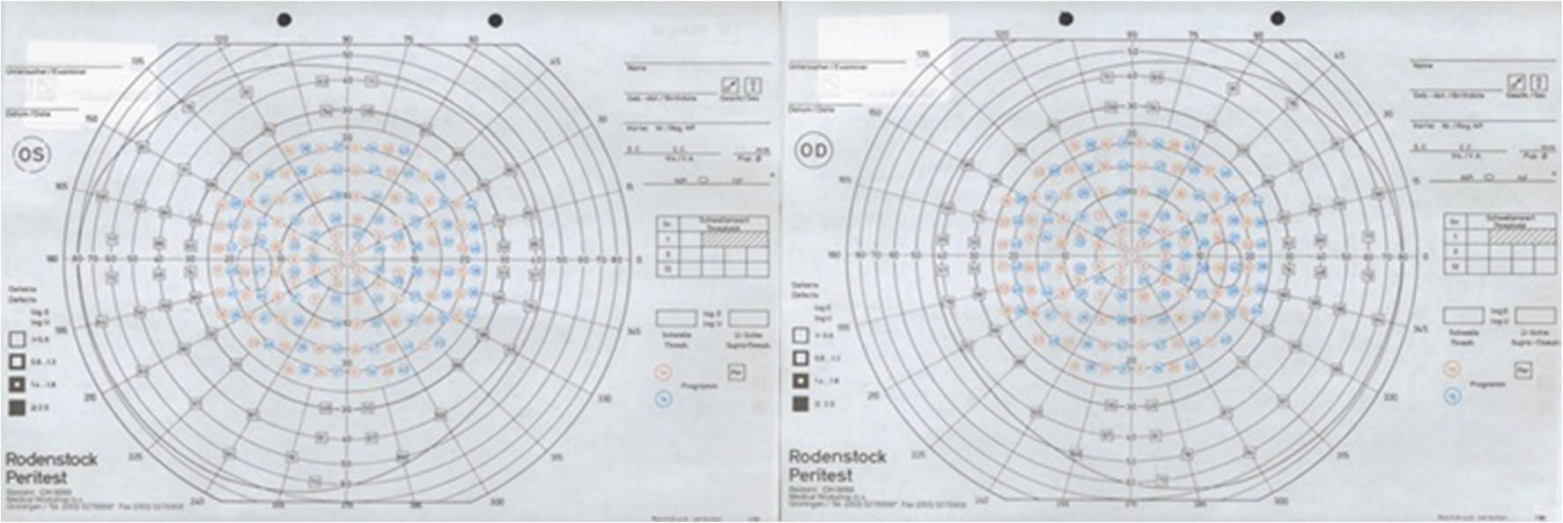
Blank Rodenstock peritest (Medical Workshop b.v., Netherlands) for the left (OS) and right (OD) eye. This static perimetry method tests a set amount of points across the visual field, where the position of the tested points is the same with every test. A grey square is printed over the tested point when the patients show VFD in the corresponding point. The Rodenstock perimetry test takes into the account the importance of central vision over peripheral vision by assessing more points in the central area of vision, especially within the central 30°

Different methods for evaluating VFD are available and can be divided into kinetic and static perimetry methods. The Rodenstock Peritest static perimetry (Medical Workshop b.v., Netherlands), shown in Fig. 2, is mostly used to determine postoperative VFD after ATL in our hospital. However, other types of static perimetry were also used by other hospitals, such as the Humphrey Field Analyser full field 120 point screening test (Carl Zeiss Meditec, Germany). The different static perimetry methods all assess a set amount of points across the visual field.

A Rodenstock perimetry scoring chart was digitally put over a grid in Microsoft Excel (Microsoft, Office, 2013), giving the different assessed points of the perimetry a coordinate which corresponds to that point. Other perimetry charts were also digitally put over this Excel grid, creating corresponding coordinates to the Rodenstock perimetry coordinates. This was done for all pre- and postoperative perimetry examinations of the included patients. By subtracting the preoperative from the postoperative perimetry, the difference in VFD following surgery became evident. By combining the perimetry charts from all included patients, an average perimetry chart is created. Each coordinate can be given a percentage, which represents the percentage of patients that showed visual field deficit on that coordinate.

All the coordinates together, with deficit percentages in one group, can be added to create an average visual field for that group. This visual representation allows for comparison between groups and demonstrates the exact VFD location. The total amount of deficit coordinates is also calculated for a quadrant or total visual field, giving an exact percentage of the visual field deficit per patient or for the whole group. Calculating the average amount of lost coordinates per group enables statistical analysis of VFD between groups. Moreover, we have analysed differences in VFD between eyes, with both resection size groups combined.

The Rodenstock peritest is by default divided into 12 equal parts of 30° each (pies), as can be seen in Fig. 2 (thick lines running to the centre). By analysing the VFD per pie, the percentage of visual field deficit in each pie of 30° becomes visually apparent. This allows to determine VFD in nasal and temporal parts of the visual field.

### Statistical analysis

Statistical analysis of categorical variables was carried out using the chi-squared and Fisher’s exact tests; comparison of means was carried out using *t* test, Wilcoxon rank sum or Mann-Whitney *U* test, after testing for normality. Analysis of resection size and VFD was conducted using linear regression analysis, only presenting the slope of the function for significant correlations. VFD is expressed as the percentage (± standard deviation) loss of visual field of the contralateral upper quadrant, the other three quadrants or the total visual field. Only *p* values < 0.05 were considered statistically significant. All analyses were conducted using IBM SPSS version 23.

## Results

### Patients

A total of 55 patients, 25 (45.5%) male, were identified and their characteristics are presented in Table 2. Twenty-five (45.5%) patients underwent right-sided ATL. Mean age was 38.9 years (range 15–62 years). Patients in the ≥ 45 mm group had a significantly higher resection length (AP) and ATOP ratio, as expected (*p* < 0.01). The exact ATOP distance differed significantly between right- and left-sided ATL. However, for analysis, we used the ratio between AP and ATOP, which showed no significant difference between both groups.

**Table 2:** Right- and left-sided surgery characteristics

Characteristic	Right-sided surgery No. (mean, range), *n* = 25	Left-sided surgery No. (mean, range), *n* = 30	*p* value
Resection size (AP^†^)	43.1 (28–80)	42.2 (11–54)	0.72
ATOP	124.1 (116–134)	128.0 (117–138)	0.01
Ratio AP-ATOP < 45 mm group	0.29 (0.21–0.33)	0.29 (0.09–0.37)	0.87
Ratio AP-ATOP ≥ 45 mm group	0.42 (0.36–0.65)	0.38 (0.34–0.41)	0.11

*AP* anterior-posterior resection distance in millimetres, *ATOP* anterior temporal-occipital pole distance in millimetres

### Perimetry

**Fig. 3:**
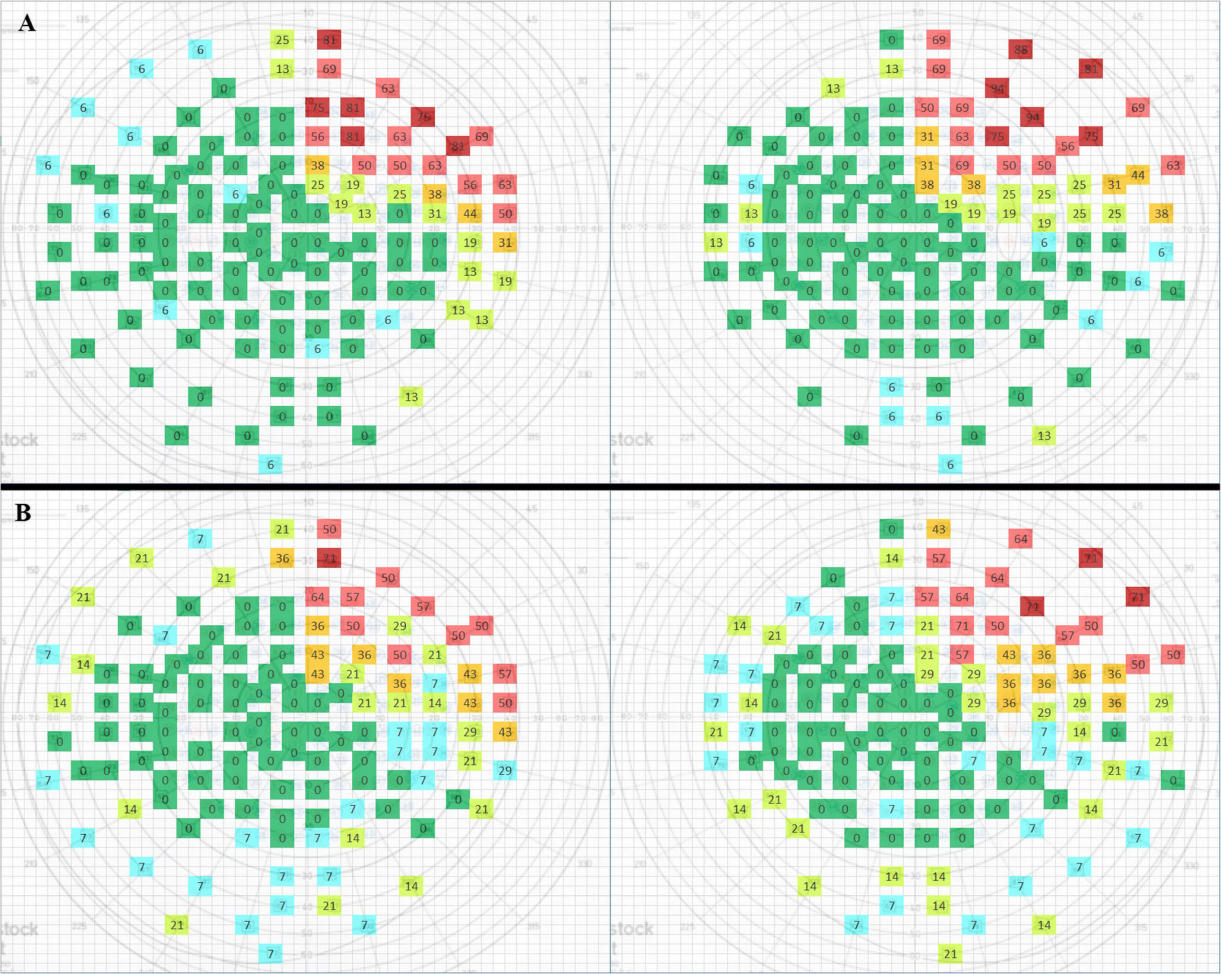
**a** For < 45 mm left-sided surgery. **b** For ≥ 45 mm left-sided surgery. VFD within the right upper quadrant is comparable. A higher amount of VFD is notable in the other three quadrants in the ≥ 45 mm group. Colours have been given to the coordinates to allow for easier visual recognition: green = no patients with visual loss at the corresponding coordinate (0%); blue = sporadic field loss (1–9% of patients); yellow = some visual field loss (10–29% of patients); orange = moderate visual field loss (30–49% of patients); light red = moderate-severe visual field loss (50–69% of patients); dark red = severe visual field loss (≥ 70% of patients)

**Fig. 4:**
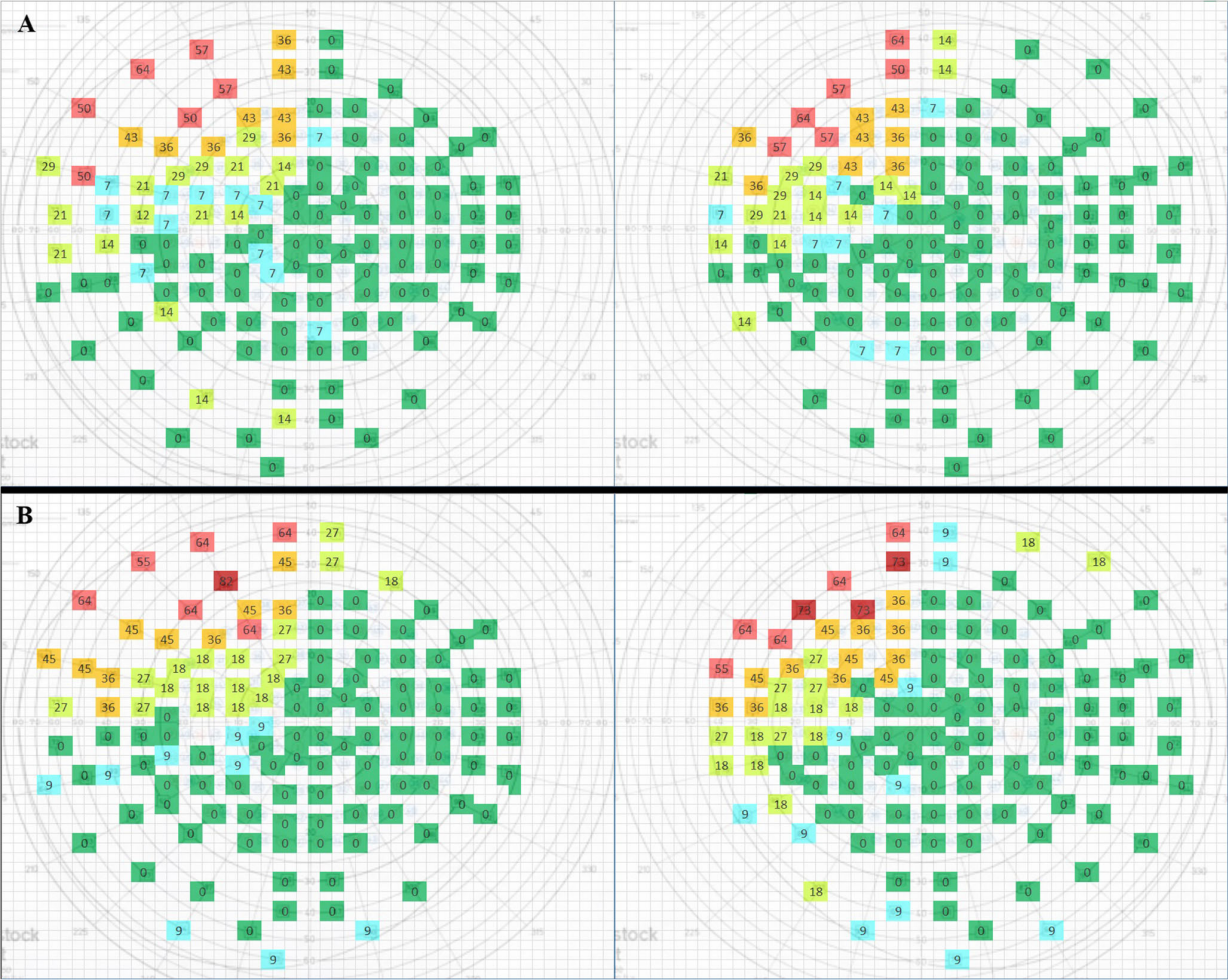
**a** For < 45 mm right-sided surgery. **b** For ≥ 45 mm right-sided surgery. VFD within the left upper quadrant is comparable. A higher amount of VFD is notable in the other three quadrants in the ≥ 45 mm group, especially in the areas bordering the left upper quadrant. Colours have been given to the coordinates to allow for easier visual recognition: green = no patients with visual loss at the corresponding coordinate (0%); blue = sporadic field loss (1–9% of patients); yellow = some visual field loss (10–29% of patients); orange = moderate visual field loss (30–49% of patients); light red = moderate-severe visual field loss (50–69% of patients); dark red = severe visual field loss (≥ 70% of patients)

A total of 39 (70.9%) patients underwent the Rodenstock static peritest, mostly in our hospital, whereas the remaining 16 patients (29.1%) underwent the Humphrey full field 120 point screening test in other hospitals. A total of 49 patients (89.1%) showed some degree of new VFD after surgery. Six patients showed no increase in postoperative VFD, including three patients in the ≥ 45 mm group. Results for the < 45 and ≥ 45 mm resection groups and left- and right-sided surgeries are shown in Figs. 3 and 4.

**Fig. 5:**
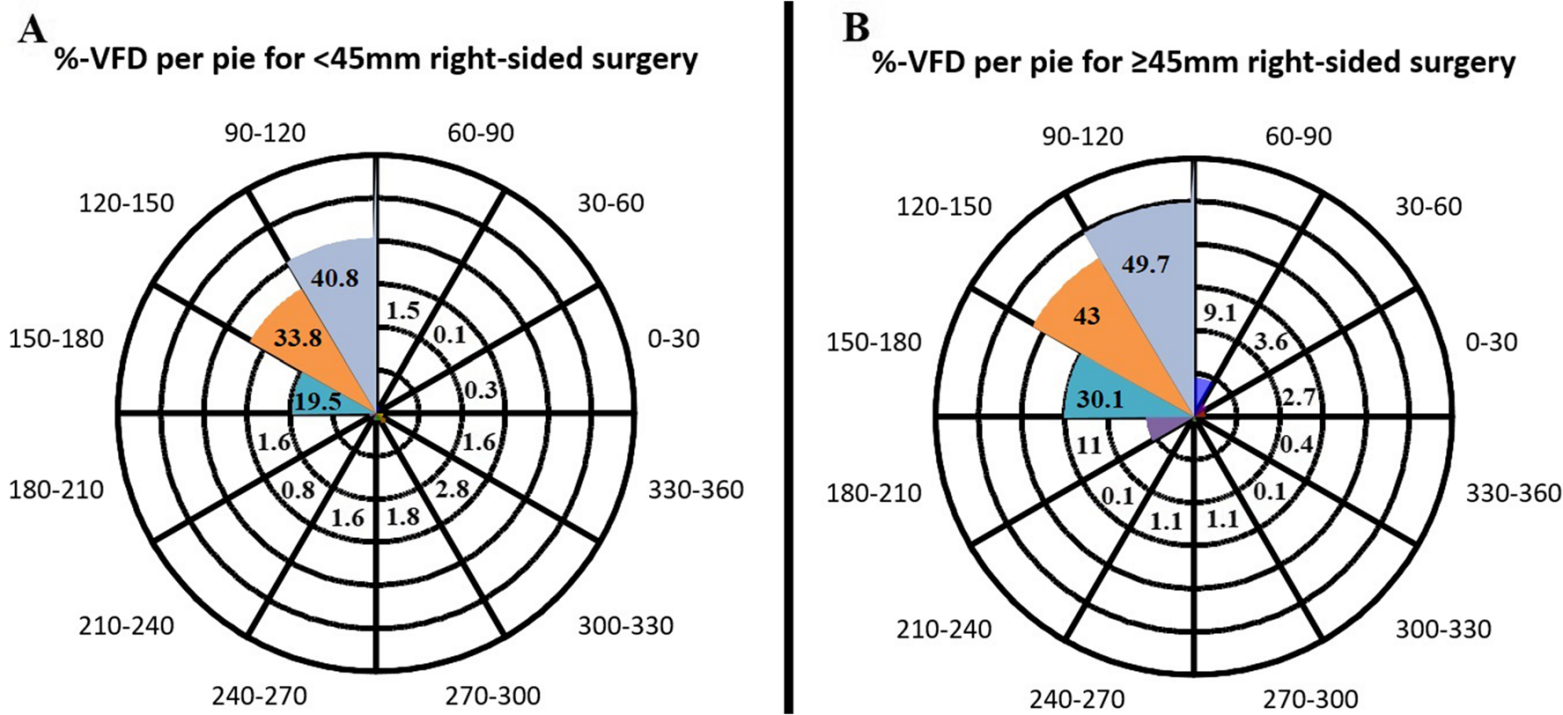
Percentage VFD per pie for right-sided surgery. **a** For the < 45 mm group. **b** for the ≥ 45 mm group. Each concentric layer represents 10% average VFD for the < 45 or ≥ 45 mm groups. The number in each pie represents the average percentage VFD in the corresponding pie. The medial pie, running from 90° to 120°, shows the largest visual defect followed by the pie from 120° to 150° and the pie running from 150° to 180°. Also, an increase in VFD in the ≥ 45 mm group is noted in the pies adjacent to the contralateral upper quadrant (60–90° and 180–210° pies)

**Fig. 6:**
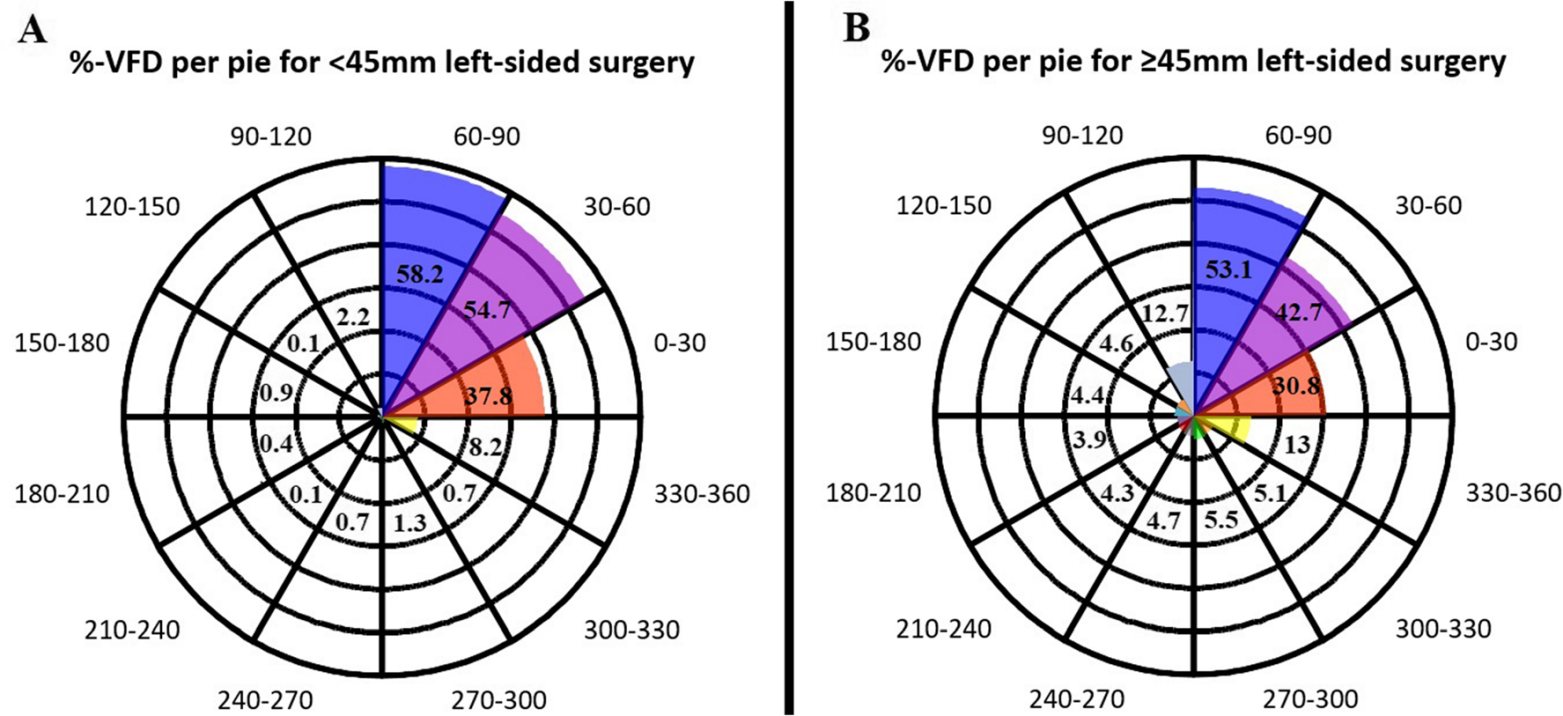
Percentage VFD per pie for left-sided surgery. **a** For the < 45 mm group. **b** For the ≥ 45 mm group. Each concentric layer represents 10% average VFD for the < 45 or ≥ 45 mm groups. The number in each pie represents the average percentage VFD in the corresponding pie. Most VFD was found in the upper contralateral medial pie running from 60° to 90°, followed by the 30–60° and 0–30° pies. Also, an increase in VFD in the ≥ 45 mm group is noted in the pies adjacent to the contralateral upper quadrant (90–120° and 330–360° pies)

The VFD for pies of 30° is illustrated in Figs. 5 and 6. Most VFDs were found in the nasal part of the contralateral upper visual quadrant of both eyes, expanding laterally towards the horizontal axis for both right- and left-sided ATL.

### Visual field deficit (Tables 3, 4 and 5)

Mean percentage VFD in the total visual field of both eyes for both right- and left-sided ATL was higher in the ≥ 45 mm group. This is in contrast to the VFD in the contralateral upper quadrant, which was higher in the < 45 mm group for left-sided surgery, though both were not statistically significant. A statistically significant larger VFD was noted for the ≥ 45 mm group compared to the < 45 mm group in the other three quadrants for right-sided surgery (0.7 ± 2.4 vs. 2.3 ± 4.4%, *p* = 0.04), but not for left-sided surgery (*p* = 0.11).

**Table 3:** Visual field deficits by side of surgery for the total visual field, contralateral upper quadrant, or other three quadrants

Side of surgery	Visual field deficit % (SD)		*p* value
	< 45 mm group	≥ 45 mm group	
Right side			
Total visual field	6.7 (6.7)	12.5 (11.7)	0.14
Left upper quadrant	28.9 (25.7)	42.9 (36.3)	0.27
Other three quadrants	0.7 (2.4)	2.3 (4.4)	0.04
Left side			
Total visual field	13.1 (7.0)	14.3 (10.9)	0.73
Right upper quadrant	48.7 (27.3)	41.2 (31.6)	0.49
Other three quadrants	1.8 (1.8)	5.7 (9.2)	0.11

An increase in VFD is noted in the ≥ 45 mm group for both right- and left-sided surgeries, but was not statistically significant. VFD within the contralateral quadrant showed no significant difference between the groups. Also, VFD outside the contralateral upper quadrant (other three quadrants) was higher in the ≥ 45 mm groups, but was only significant for right-sided surgery

**Table 4:** Comparison of VFD per eye by side of surgery for the total visual field, contralateral upper quadrant, or other three quadrants

Side of surgery	Visual field deficit % (SD)		*p* value
	Right eye	Left eye	
Right side			
Total visual field	8.8 (9.9)	9.7 (9.7)	0.41
Left upper quadrant	33.1 (30.3)	37.3 (33.5)	0.18
Other three quadrants	1.0 (3.5)	0.4 (4.7)	0.45
Left side			
Total visual field	14.5 (9.8)	12.9 (8.3)	0.03
Right upper quadrant	45.5 (29.4)	45.0 (29.7)	0.79
Other three quadrants	3.3 (7.1)	3.9 (6.5)	0.39

Our study demonstrates more VFD in the contralateral eye for both right- and left-sided surgeries, but was only statistically significant for left-sided surgery. VFD in the contralateral upper quadrant, or other three quadrants combined showed no significant difference between the right and left eye

**Table 5:** Visual field deficit per side of surgery for all patients, < 45 mm group, and ≥ 45 mm group

	Visual field deficits per side of surgery % (SD)		*p* value
	Right	Left	
Groups			
Both groups	9.3 (9.5)	13.7 (8.9)	0.08
< 45 mm	6.7 (6.7)	13.1 (7.0)	0.02
≥ 45 mm	12.5 (11.7)	14.3 (10.9)	0.70

When comparing VFD between sides of surgery, we showed more VFD for left-sided surgery, though only statistically significant in the < 45 mm group

We also analysed VFD for each separate eye, combining the < 45 and ≥ 45 mm groups. As shown in Table 4, the contralateral eye showed more VFD than the ipsilateral eye, though this was only found significant for left-sided ATL (14.5 ± 9.8 vs. 12.9 ± 8.3%, *p* = 0.03). No significant difference was demonstrated in the contralateral upper quadrant or other three quadrants.

When comparing VFD by side of surgery, VFD in both eyes was larger for patients with left-sided surgery, for the < 45 mm group, ≥ 45 mm group, and both groups combined. This was only statistically significant in the < 45 mm group (6.7 ± 6.7 (right-sided ATL) vs. 13.1 ± 7.0% (left-sided ATL), *p* = 0.02), as is shown in Table 5.

### Resection size and VFD

**Fig. 7:**
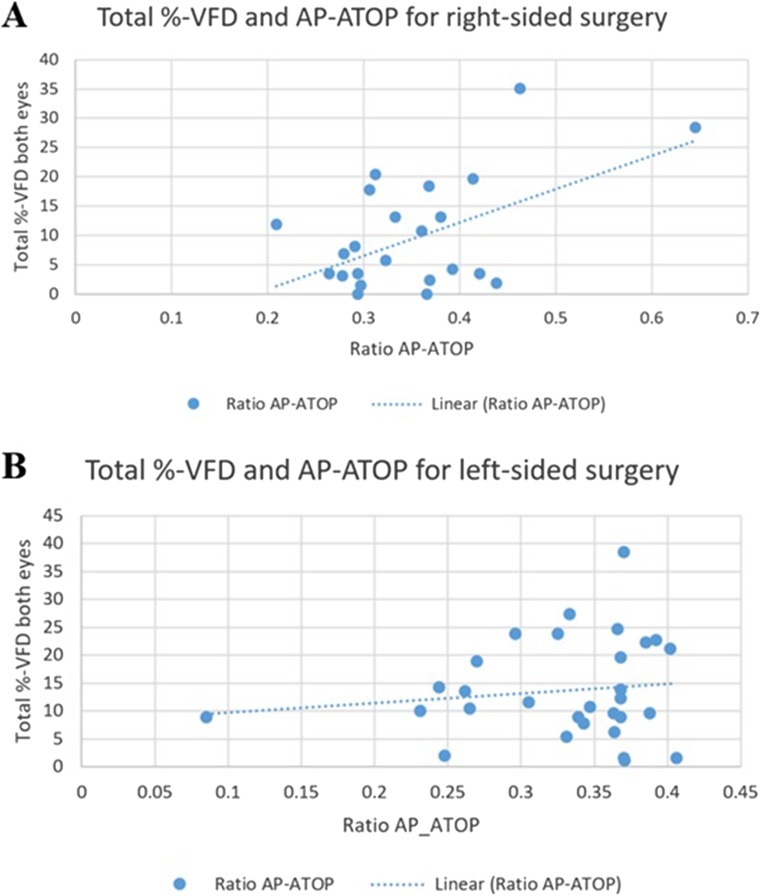
Linear regression modelling between total percentage VFD for both eyes combined and AP-ATOP ratio. **a** A statistically significant correlation was demonstrated for right-sided surgery. Two outliers were noted (ratio AP-ATOP 0.463, VFD 35%; ratio 0.645, VFD 28%), excluding any of these outliers we again noted a significant linear correlation. **b** Linear regression modelling between total percentages VFD for both eyes combined and AP-ATOP ratio for left-sided surgery showed no statistically significant correlation

First, we assessed the correlation between the AP-ATOP ratio and the percentage of total VFD in both eyes in patients who underwent right-sided ATL. A significant correlation between ratio AP-ATOP and VFD for right-sided surgery was found (*r* = 0.52, *p* < 0.01). Linear regression modelling showed a 0.57% increase in VFD of the total visual field of both eyes for every 0.01 (on average equal to 1.25 mm) increase in AP-ATOP ratio (95% CI 0.016–0.97, Fig. 7a). A similar linear regression model was performed for patients following left-sided ATL. Here, the total VFD of both eyes was not found significantly correlated (*p* = 0.49) (Fig. 7b).

## Discussion

In this study, a novel quantitative scoring method in static perimetry was developed to assess VFD in patients who underwent a temporal lobectomy for the treatment of drug-resistant epilepsy. This new method enables the assessment of a more realistic estimate of the amount of VFD after ATL, allows statistical analysis, and is directly clinically applicable. Using this method, we demonstrated that following temporal lobectomy, most VFDs are located in the contralateral upper quadrant, which can even extend beyond the vertical and horizontal borders of the quadrant.

### Quantifying VFD using various perimetric methods

Our study demonstrates that this novel quantification procedure can be applied for the evaluation of perimetric results acquired by different perimetry methods, facilitating easy and reliable comparison. The different perimetry methods included in this study are easily translatable into the Rodenstock charts, so that the proposed method is equally applicable for all methods. Moreover, we estimate that this method can also be applied to quantify VFD for patients with kinetic perimetry, by translating the borders of vision into corresponding coordinates on a Rodenstock perimetry chart, making it a universal method for the quantitative assessment of VFD after ATL. As the described study cohort did not include patients with kinetic perimetry, this will be a purpose for future studies.

### Localising VFD (contralateral upper quadrant, nasal vs. temporal pies)

The main role of the optic radiation is to convey visual information from the lateral geniculate nucleus (LGN) to the primary visual cortex. The anterior part of the Meyer’s loop contains fibres corresponding to the nasal part of the contralateral upper quadrant, whereas the fibres in the posterior part correspond to the temporal part of the upper quadrant [1]. Because the anterior portion of ML is more likely to be injured after temporal lobe resection, visual field deficits after ATL will cause more frequently nasal than temporal VFD’s of the contralateral upper quadrant. This is indeed confirmed by our results. Sometimes the temporal sector can even be spared, leading to an incomplete contralateral superior quadrantanopia. We found that complete Meyer’s loop injury results in a complete upper quadrant VFD (Figs. 3, 4, 5 and 6). This is in line with the observations of previous studies [1, 8, 13, 31].

### Effect of resection length

As mentioned, the distance from the anterior tip of Meyer’s loop to the anterior tip of the temporal pole has been reported to range from 22 to 44 mm, suggesting that all patients in the ≥ 45 mm group should have some degree of VFD. However, 3 of 25 patients with a resection length ≥ 45 mm (48, 49 and 51 mm) had no VFD. This suggests that the anterior tip of ML was located more posteriorly than 45 mm in these patients. In general, increasing resection length leads to increasing VFD due to a larger injured portion of ML. In contrast, we found a larger percentage of VFD in the contralateral upper quadrant in the < 45 mm group compared to the ≥ 45 mm group, for both left- and right-sided ATL, although this difference was not statistically significant. This may be the result of interindividual differences in ML.

Furthermore, more VFDs in the other three quadrants was noted in the ≥ 45 mm group compared to the < 45 mm group. To the best of our knowledge, no studies reported on the quantification or analysis of VFD outside the contralateral upper quadrant. Barton et al. states that linear regression suggested “involvement of the lower quadrant when resections reached 70 to 79 mm” [1]. However, our study evidently point out involvement of the lower quadrant with various resection lengths as low as 28 mm. This emphasises the difficulty to predict postoperative VFD in individuals, due to wide interindividual variability in the anatomy of ML. It has been indicated that preoperative diffusion tensor imaging (DTI) tractography of the visual pathways can reduce the incidence of postoperative VFD [30]. Therefore, combining a DTI MRI before surgery with postoperative VFD calculations might produce interesting results, which can also be a topic of future VFD quantification studies.

### Differences between left- and right-sided surgeries

DTI studies assessing the anatomy of Meyer’s loop found a trend towards a more anterior extending left optic radiation [10, 12, 30, 31]. Jeelani et al. described that VFD’s were 3.5 times more likely following left-sided ATL [13]. This is reflected by the higher incidence of VFD, particularly in the smaller resection lengths (< 45 mm), in our patients with left-sided ATL. This is further emphasised by the on average larger resections performed on the non-dominant right side. Literature on whether the resection length is a significant predictor of postoperative VFD is non-conclusive, even though most research found a significant correlation between resection lengths and postoperative VFD [1, 13, 24, 31]. In concordance with most papers we also found a significant correlation between resection length (AP-ATOP distance) and VFD, although only for right-sided surgery. The suggestion of a more anterior located ML in the left hemisphere might explain why we did not find this relation for left sided ATL. As a consequence, a smaller resection length in the left hemisphere causes relatively more damage to ML, resulting in more VFD, while on the right side, there is a more gradual increase in VFD with resection size.

Additionally, we found significantly more VFD’s in the right eye compared to the left eye following left-sided ATL. A similar trend was noted following right-sided surgery, with more VFD in the contralateral eye. However, studies reporting on the anatomical topology of the ipsilateral and contralateral fibres within Meyer’s loop are lacking. Our findings suggest a more anterior location of the contralateral eye fibres in Meyer’s loop compared to the ipsilateral fibres, making the contralateral eye more susceptible to injury by ATL, resulting in more VFD.

### Clinical relevance

The clinical consequences of the presence of VFD’s following surgery were not assessed. Assessment should include impact on quality of life and consequences for the eligibility to drive. Clinical experience learns that patients who underwent temporal lobe resection rarely complain of visual field loss. Hensley-Judge et al. stated that “the quality of life is not adversely affected by the presence of postsurgical VFD when defects are limited to the upper quadrant” [9]. In our study, we did find a significant increase in VFD outside the contralateral upper quadrant, particularly into the inferior quadrant. The clinical significance of this finding has yet to be determined, as well as the clinical importance of increased VFD’s with increasing resection lengths, even beyond the borders of the contralateral upper quadrant.

Most patients cite the ability to drive as one of the five most important factors that would contribute to their complete rehabilitation from epilepsy [25]. However, in 25–46% of patients, the VFD may be severe enough to fail the current visual field criteria set by the UK Driver and Vehicle Licensing Authority (DVLA), even when patients are seizure free postoperatively [17, 18, 22]. Since the regulations for driving with visual impairment are the same in the UK and the Netherlands, comparable percentages are to be expected in the Netherlands [3, 27]. So, examining eligibility to drive after ATL could be an interesting translation to clinical relevance of VFD.

## Limitations and future perspectives

Clinical impact of postoperative VFD was beyond the scope of this study. This poses a challenge for future research. Quality of life questionnaires related to visual complaints are only extensively used and validated for glaucoma patients [4, 28]. However, the pattern of visual field loss in these patients is different from VFD following ATL. Also, most patients with glaucoma are evaluated using Humphrey 24-2 or 30-2 perimetry, assessing only the central field of vision. In order to adequately determine clinical significance of postsurgical VFD, existing validated quality of life questionnaires should be complemented with specific ophthalmological questions for these patients.

Most perimetry scoring methods only assess whether a coordinate has been seen or not. Even though these methods assess more coordinates in the central field of vision, more sophisticated calculations that better approximate physiological central magnification and more precise perimetric assessments of the depth of field loss might further improve the correlation. In some perimetry scoring methods, whether a point is seen or not is not dichotomous. Our proposed method does not take this into account, resulting in a potential information loss when translating between the methods. Perhaps further developments and research can take the depth of field loss into account.

The patients collected in this study were retrospectively recruited form a prospectively collected epilepsy surgery database. We decided to only include patients with complete pre- and postoperative imaging and perimetry data which resulted in the total of 55 patients, out of 89 surgically treated patients. Given the retrospective design, the potential for recall and selection bias is present.

In our study, none of the included patients underwent VFD evaluation using kinetic perimetry. We believe that translating kinetic into static perimetry as a universal scoring method is easily performed, using the proposed method as earlier described. Future studies applying this method on kinetic perimetry data will point out feasibility of our method for kinetic perimetry.

While the included number of patients is relatively large compared to other similar studies, we divided patients in groups based on resection length and side of surgery. We first attempted to combine right- and left-sided surgeries, but large differences between both sides of surgery became apparent. Therefore, subgroups were created, reducing the power of statistical analysis. Nevertheless, this study demonstrated statistical significance for right-sided surgery. Also, we noted the relatively large standard deviation in VFD, making it hard to make recommendations and underlining the large interindividual dispersion of postoperative VFD. Including more patients will improve the statistical power and significance.

## Conclusion

In this study, we have developed a novel quantitative scoring method for the assessment of postoperative visual field deficits and assessed its feasibility for clinical use. Moreover, when applying this method in patients who underwent temporal lobe resection, a significant correlation for resection length and postoperative VFD in right-sided ATL was demonstrated, with VFD beyond the borders of the contralateral upper quadrant. Clinical relevance of these VFD is to be assessed in a future study in this patient group.

## Abbreviations

ATL: Anterior temporal lobectomy
TLE: Temporal lobe epilepsy
AH: Amygdalohippocampectomy
VFD: Visual field deficit
ML: Meyer’s loop
MRI: Magnetic resonance imaging
AP: Anterior-posterior resection distance
ATOP: Anterior temporal-occipital pole distance
SD: Standard deviation
